# ﻿The revalidation of *Otostigmus* (*O.*) *lewisi* Song et al., 2005 (Scolopendromorpha, Scolopendridae) based on new material from Jiacha County, China

**DOI:** 10.3897/zookeys.1088.77703

**Published:** 2022-03-04

**Authors:** Xiaoshuang Liu1, Yixuan Li*, Zhiyong Di

**Affiliations:** 1 Key Laboratory of Zoological Systematics and Application of Hebei Province, College of Life Sciences, Hebei University, Baoding, Hebei 071002, China Hebei University Baoding China; 2 Institute of Life Science and Green Development, Hebei University, Baoding, Hebei 071002, China Hebei University Baoding China

**Keywords:** Centipede, *Otostigmusberoni*, *Otostigmuslewisi*, taxonomy, Tibet, Xizang

## Abstract

Otostigmus (O.) lewisiSong et al., 2005 was described from sub-adult specimens from Jiacha County (Xizang, China), but was synonymized by Lewis (2010) with the Nepalese species O. (O.) beroni Lewis, 2001. The latter was also recorded from Jilong County (Xizang, China) by Song et al. (2005). Following a comparison of O. (O.) beroni from Jilong County with new materials of O. (O.) lewisi from Jiacha County, we reaffirm that O. (O.) lewisi is a valid species.

## ﻿Introduction

The subgenus Otostigmus Porat, 1876 currently comprises about 56 species (Bonato et al. 2016; Niu et al. 2021). Among them, eight have been recorded in China (Song et al. 2005; Bonato et al. 2016; Niu et al. 2021): O. (O.) aculeatus Hasse, 1887, O. (O.) astenus (Kohlrausch, 1878), O. (O.) beroni Lewis, 2001, O. (O.) lewisi Song, Gai, Song & Zhu, 2005, O. (O.) martensi Lewis, 1992, O. (O.) politus Karsch, 1881, O. (O.) scaber Porat, 1876, and O. (O.) xizangensis Niu, Li & Di, 2021.

Otostigmus (O.) lewisi was identified as a synonym of O. (O.) beroni (Lewis 2010). According to the original description, O. (O.) lewisi has complete paramedian sutures on sternites 4–19, sternite 21 has a shallow medial longitudinal depression, and the coxopleural process is short. However, the sternite paramedian sutures in O. (O.) beroni are incomplete, and the coxopleural process is long. These differences were not shown in the corresponding figures of Song et al. (2005). Thus, Lewis (2010) wrote: “There are no significant differences between the two species and *O.lewisi* is a junior subjective synonym of *O.beroni*”. We would also like to highlight that all type materials used in the Song et al. (2005) of O. (O.) lewisi were sub-adults.

## ﻿Materials and methods

Studied materials were collected by hand and preserved in 75% ethanol. All studied materials in this paper were examined under a stereomicroscope (Motic K700). Photographs and measurements were taken using a Leica Stereomicroscope (M205 A). The standard terminology followed Bonato et al. (2010). The repository acronym is MHBU (Museum of Hebei University, Baoding, China).

## ﻿Results

### ﻿Order Scolopendromorpha Leach, 1814

#### Family Scolopendridae Leach, 1814

**Subfamily Otostigminae Kraepelin, 1903**

**Genus *Otostigmus* Porat, 1876**

#### Subgenus Otostigmus Porat, 1876

##### ﻿ Otostigmus (O.) beroni

Taxon classificationAnimalia

Lewis, 2001

60ED176E-3416-5BCE-9AFD-79035CE69070

Figs 1 , 4C

Otostigmus beroni Lewis, 2001: 22; Lewis 2010: 26.

###### Material examined.

Ar.-MHBU-SoJL1608050101–Ar.-MHBU-SoJL1608050120: Jilong Town, Jilong (Gyirong) County, Xizang (Tibet), China, 28.4370°N, 85.2568°E, 5/8/2016, leg. Zhiyong Di. Ar.-MHBU-SoJL21080101: Jilong Town, Jilong (Gyirong) County, Xizang (Tibet), China, 28.4350°N, 85.2570°E, 1/8/2021, leg. Zhiyong Di. Housed in MHBU.

###### Diagnosis.

**Figure 1.**
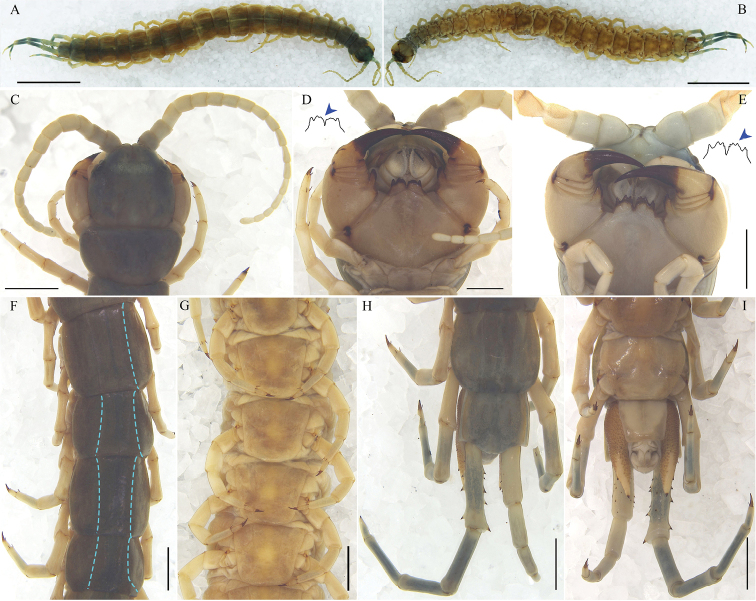
Otostigmus (O.) beroni Lewis, 2001 **A, B, G** Ar.-MHBU-SoJL1608050101 **C, D, F, H, I** Ar.-MHBU-SoJL1608050104 **E** Ar.-MHBU-SoJL1608050105 **A** dorsal view **B** ventral view **C** cephalic plate, antennae, tergite 1 and legs 1–2, dorsal view **D, E** ventral view of head, basal antennal articles, an arrow showing the profile of the tooth plate **F** tergites 16–19 **G** sternites 11–14 **H** last two segments and ultimate legs, dorsal view **I** last two segments and ultimate legs, ventral view. Scale bars: 10.0 mm (**A, B**); 2.0 mm (**C, F–I**); 1.0 mm (**D, E**).

Maximum length 57 mm. Antennae 18 articles, the basal 2.25 to 2.40 glabrous dorsally (Fig. 1C, E). With 4 or 5 teeth (rarely 3) on each tooth plate, the median 3 (or 4) ones more or less fused (Fig. 1D, E). Sternites with incomplete paramedian sutures occupying at least anterior 66 to 100% in mid and hind body region (Fig. 1G). Coxopleural process long, typically with 2 apical spines, 1–2 lateral spines and 1–2 dorsal spines (Fig. 1I). Ultimate prefemur with 4 rows of prominent spines, disposed on swollen bases (10–16 spines in total) (Fig. 1H, I).

###### Description

**(Ar.-MHBU-SoJL1608050101). *Length***: 54 mm (measured from anterior margin of cephalic plate to posterior margin of tergite 21).

***Pigmentation*** (after remaining in alcohol for five years): body color yellow-brown; legs and antennae yellow; cephalic plate, tergites 1–2, tergites 20–21 and penultimate and ultimate legs blue-green (Fig. 1A, B). Live individual (Ar.-MHBU-SoJL21080101): cephalic plate blue-green, tergites blue-brown, penultimate legs and ultimate legs yellow with blue middle part in each segment, the other legs yellow (Fig. 4C).

***Cephalic plate***: wider than long, rounded anteriorly, without sutures or sulci (Fig. 1C).

***Antennae***: 18 articles, basal 2.3 glabrous, the remainder are covered with short hairs (Fig. 1C, E).

***Forcipular segment***: tooth plates with 4 teeth in each plate, the median 3 fused. Process of forcipular trochanteroprefemur with 3 teeth (Fig. 1D).

***Tergites (T)***: with complete paramedian sutures from 3 to 20, marginate from 7 to 21 (Fig. 1F). Central part of the posterior border of T21 slightly convex (Fig. 1H).

***Sternites (S)***: smooth, with incomplete paramedian sutures from 5 to 19 (Fig. 1G); S21 with a slight median longitudinal depression and converging posteriorly (Fig. 1I).

***Coxopleuron***: with numerous small pores, coxopleural process moderately long and apical border with protuberance (Fig. 1I). With pore-free longitudinal strips within pore field. Coxopleural process with 2 apical spines, 2 lateral spines, and 1–2 dorsal spines.

***Legs (L)***: L1–16 and L18–19 with 2 tarsal spurs, L1–4, left L5 and right L6 with 1 tibial spur and L1 with 1 femoral spur.

Ultimate prefemur with 4 ventro-lateral, 2 ventro-medial, 3 medial, 2 dorso-medial spines and 1 corner spine (Fig. 1H, I).

###### Variability.

There are multiple differences among individuals as addressed below. The number of teeth of forcipular tooth plates 4+4 (11 specimens), 5+5 (6 specimens), 3+3 (2 specimen) or 3+4 (2 specimens). Coxopleural process with 4–7 spines (2 apical spines, 1–2 lateral spines and 1–3 dorsal spines). One tibial spur on L1–4 (8 specimens), L1–5 (5 specimens), L1–2 (2 specimens), L1–3 (3 specimens), or L1–6 (2 specimens). Two tarsal spurs on 1 to 18 or 19 pairs of legs; 1 tarsal spur on subsequent to penultimate legs. Ultimate legs without tarsal spur. Ultimate legs prefemur with 11–14 spines (4 or 5 ventro-lateral, 1–3 ventro-medial, 3 or 5 medial and 1 or 2 dorso-medial and 1 corner spine, rarely 0 or 3 corner spines) (Table 1).

**Table 1.** Variation in O. (O.) beroni from Jilong County, Xizang (1: Ar.-MHBU-SoJL1608050101; 2: Ar.-MHBU-SoJL1608050102; 3: Ar.-MHBU-SoJL1608050103; 4: Ar.-MHBU-SoJL1608050104; 5: Ar.-MHBU-SoJL1608050105) (L/R: Left/Right).

		Specimens				
		1	2	3	4	5
Length/mm		54	43	47	57	39
Number of antennal articles (L/R)		18/19	18	18	18	18
Number of glabrous basal antennal articles		2.3	2.3	2.4	2.4	2.3
Tooth-plate teeth		4+4	4+4	5+5	4+4	5+5
Paramedian sutures on tergites		3–20	4–20	4–20	3–20	4–20
Tergites marginate		6–21	6–21	7–21	7–21	6–21
Paramedian sutures on sternites		5–19	7–19	7–19	7–19	6–19
Coxopleural process	Apical spines (L/R)	2/3	2	2	2	2
	Dorsal spines (L/R)	1/2	1	3	2	2/1
	Lateral spines (L/R)	1/2	2	2	2/1	2
Legs	With tibial spur	1–4	1–2	1–5	1–4	1–3
	With 1 tarsal spur	17, 20	20	20	20	20
	With 2 tarsal spurs	1–16, 18–19	1–19	1–19	1–19	1–19
Spines of ultimate prefemur	Ventro-lateral (L/R)	4	5/4	4	4	5/4
	Ventro-medial (L/R)	2	1/2	2	2/3	2
	Medial (L/R)	3	5/3	3	3/5	3
	Dorso-medial (L/R)	2	2	2	2	2/1
	Corner spine (L/R)	1	3/1	1	1/0	1
Femoral spur of leg 1		0	0	1	1	1

###### Habitat.

Found under stones in humid mountain bush (Fig. 4A).

###### Distribution.

China (Xizang) and Nepal (Fig. 5).

##### ﻿ Otostigmus (O.) lewisi

Taxon classificationAnimalia

Song, Gai, Song & Zhu, 2005 (Revalidated name)

80F267B0-9D5B-5FC6-A29B-D11CC060ACBE

Figs 2 , 3 , 4D

Otostigmus lewisi Song et al, 2005: 304.

###### Material examined.

Ar.-MHBU-SoJC1908060301– Ar.-MHBU-SoJC1908060307: Jiacha County (Gyaca County), Xizang (Tibet), China, 29.0857°N, 92.3430°E, 6/8/2019, leg. Zhiyong Di. Ar.-MHBU-SoJC1608DX01: Jiacha County, Xizang, China, 29.1188°N, 92.6969°E, 12/8/2016, leg. Zhiyong Di. Ar.-MHBU-SoJC1608120401: Jiacha County, Xizang, China, 29.1387°N, 92.6880°E, 12/8/2016, leg. Zhiyong Di. Housed in MHBU.

###### Diagnosis.

**Figure 2.**
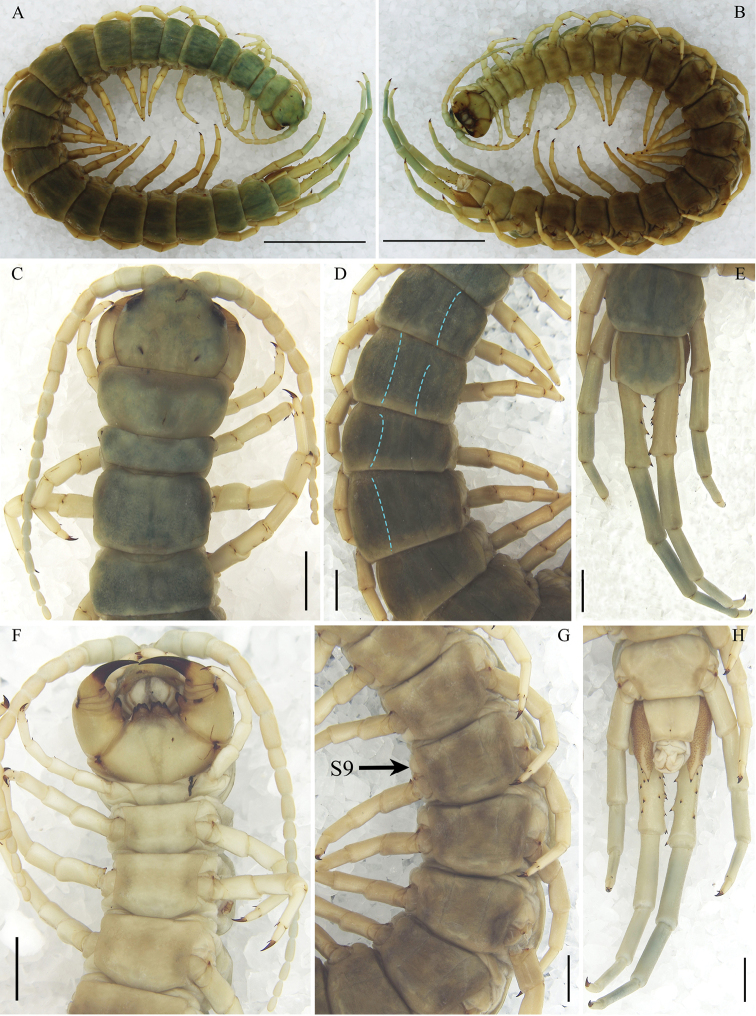
Otostigmus (O.) lewisiSong et al., 2005. (Ar.-MHBU-SoJC1908060301): **A** dorsal view **B** ventral view **C** cephalic plate, antennae and tergites 1–4, dorsal view **D** tergites 7–11 **E** last two tergites and ultimate legs, dorsal view **F** head, antennae, sternites 1–4 and teeth on tooth plates 3+3, ventral view **G** sternites **H** last two sternites and ultimate legs, ventral view. Scale bars: 10.0 mm (**A, B**); 2.0 mm (**C–H**).

**Figure 3.**
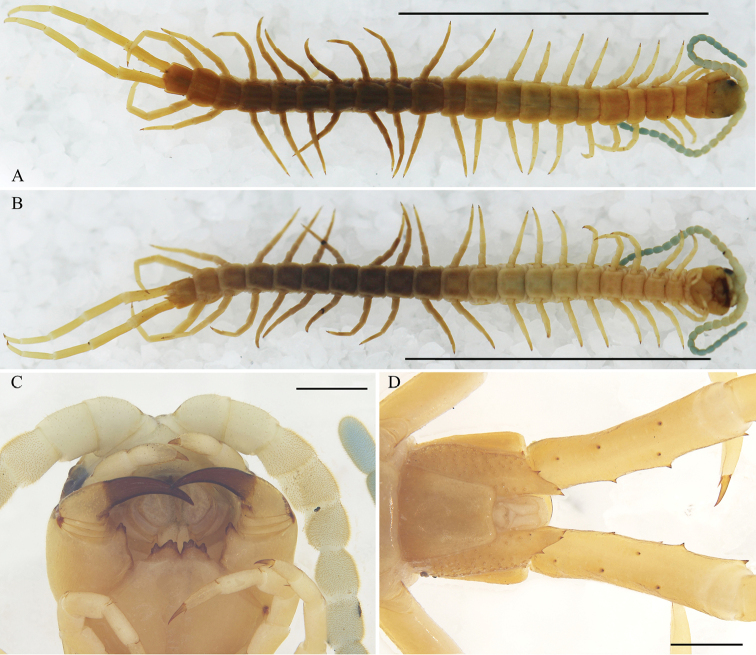
Otostigmus (O.) lewisiSong et al., 2005. (Ar.-MHBU-SoJC1908060304): Juvenile individual **A** dorsal view **B** ventral view **C** the ventral view of head, antennae and teeth on tooth plates 3+3 (the medial two teeth fused, the lateral one with a small tooth on its lateral side) **D** sternite 21, coxopleuron and prefemur of ultimate legs, ventral view. Scale bar: 10.0 mm (**A, B**); 0.5 mm (**C, D**).

Maximum length 77 mm. Antennae 17–20 articles, basal 2.2–3 glabrous dorsally (Fig. 2C, F). With 3 or 4 teeth on each tooth plate, the median two more or less fused (Figs 2F, 3C). Sternites with paramedian sutures. Coxopleural process typically with 2 apical spines, 1–2 lateral spines and 1–2 dorsal spines (Figs 2H, 3D). Ultimate prefemur with 4 rows of prominent spines, disposed on swollen bases (7–19 in total) (Fig. 2E, H).

###### Remarks.

The O. (O.) lewisi holotype described in Song et al. (2005) was as follows (Song et al. 2005; Lewis 2010): 18 articles, with the basal 2.5–2.7 glabrous dorsally; with 3 teeth on each tooth plate, the inner two ones more or less fused; tergites with complete paramedian sutures from 5; marginate from S6 to S8, without keels or spines; sternites with complete paramedian sutures from 4 to 19; last sternite with sides converging caudally, posterior edge strongly concave, and central longitudinal depression; coxopleural process short, typically with 2 apical spines, 2 lateral spines and one dorsal spine; ultimate prefemurs with prominent spines on swollen bases.

Following Song et al. (2005), Lewis (2010) considered that there were minor differences between O. (O.) lewisi and O. (O.) beroni; however, the description and figures of O. (O.) lewisi were not consistent in Song et al. (2005), as the authors mistakenly reused the figures of O. (O.) martensi (Song et al. 2005: figs 34–40) for O. (O.) lewisi (Song et al. 2005: figs 47–53). This can be verified by checking the information of O. (O.) lewisi (Song 2004: 58–59, fig. 42) and O. (O.) martensi (Song 2004: 57, fig. 40) provided in Song’s unpublished thesis.

Song et al. (2005) recorded that O. (O.) lewisi is similar to O. (O.) beroni, and distinguished O. (O.) lewisi from the latter by the length of coxopleural process and the shape of the ultimate sternite. Because Lewis (2010) didn’t refer to the unpublished thesis of Song (2004), the misleading figures of O. (O.) lewisi in Song et al. (2005) led Lewis to conclude that there are no significant differences between the two species and that O. (O.) lewisi is a junior subjective synonym of O. (O.) beroni (Lewis 2010).

The characteristics of the holotype of O. (O.) lewisi reported by Song (2004) and Song et al. (2005) are same to the new immature materials examined in this paper. Therefore, we considered that all the type materials of O. (O.) lewisi described previously were sub-adults.

###### Description

**(Ar.-MHBU-SoJC1908060301). *Length***: 70 mm (measured from anterior margin of cephalic plate to posterior margin of tergite 21).

**Figure 4.**
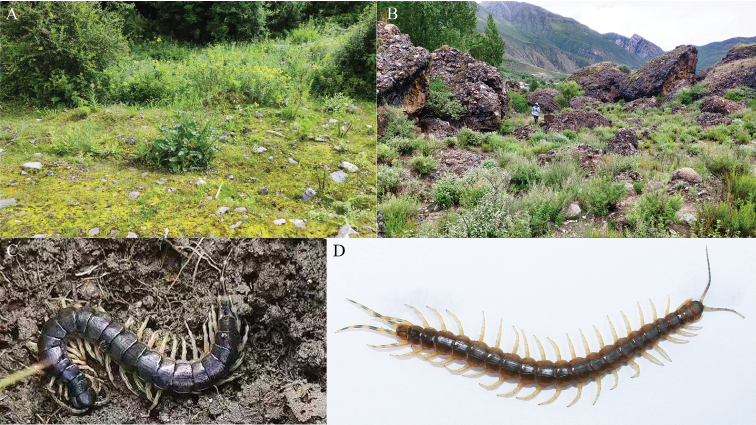
**A** habitat of O. (O.) beroni in Jilong Town, Jilong County **B** habitat of O. (O.) lewisi in Anrao Town, Jiacha County **C**O. (O.) beroni (Ar.-MHBU-SoJL21080101) **D**O. (O.) lewisi (Ar.-MHBU-SoJC1608DX01).

***Pigmentation*** (after remaining in alcohol for two years): cephalic plate and tergites yellow with light green; antennae and legs yellow; penultimate legs and ultimate legs green (Fig. 2A, B). Live individual (Ar.-MHBU-SoJC1608DX01): antennae light blue mainly, cephalic plate and tergites brownish, penultimate legs and ultimate legs yellow with blue middle part in each segment, the rest of the legs yellow (Fig. 4D).

***Cephalic plate***: wide 3.86 mm, long 3.18 mm, rounded anteriorly, without sutures or sulci (Fig. 2C).

***Antennae***: with 17 articles on the right and 19 on the left antenna, basal 2.5 glabrous, the remainder covered with short, tapering, yellowish hairs (Fig. 2C, F).

***Forcipular segment***: forcipular tooth plates present, with 3 teeth on each plate, the median two fused, their basal sutures form right angle, process of forcipular trochanteroprefemur well developed (Fig. 2F).

***Tergites (T)***: with complete paramedian sutures from 4 to 20; marginate from 6 to 21 (Fig. 2D); the posterior border of T21 slightly convex (Fig. 2E).

***Sternites (S)***: smooth, with incomplete paramedian sutures from 3 to 4, complete paramedian sutures from 5 to 19 (Fig. 2G); S21 with a slight median longitudinal depression and converging posteriorly (Fig. 2H). Central part of the posterior border of S21 slightly concave.

***Coxopleuron***: pore field with numerous pores, coxopleural process moderately long and apical border with protuberance. With pore-free longitudinal strip in pore field. Coxopleural process with 2 apical spines, 1 lateral spine and 1–2 dorsal spines (Fig. 2H).

***Legs (L)***: L2–9, right L11 and left L12 with 2 tarsal spurs; 1 tarsal spur on subsequent to penultimate legs; L1 and left L2 with 1 tibial spur and right L1 with 1 femoral spur.

The left prefemur with 1 corner spine, 3 ventro-lateral spines, 1 ventro-medial spines, 2 medial spines, 1 dorso-medial spine; the right prefemur with 2 corner spines, 5 ventro-lateral spines, 2 ventro-medial spines, 7 medial spines, 3 dorso-medial spines (Fig. 2E, H).

###### Variability.

Adult and juvenile individuals differ primarily in body length and pigmentation (Figs 2A, B, 3A, B). There are differences among individuals as described below. Antennal articles 17–20. The number of teeth of forcipular tooth-plates 3+3 (5 specimens) (Fig. 2F) or 4+4 (the lateral one with a small tooth on its lateral side) (4 specimens) (Fig. 3C). Tergites with paramedian sutures from 3 (3 specimens) or 4 (5 specimens), from 6 (1 specimen), marginate from 3–8. Sternites with paramedian sutures from 2 (4 specimens), 3 (4 specimens) or 4 (1 specimen). Coxopleuron process with 4–6 spines (2 apical spines, 1–2 lateral spines and 1–2 dorsal spines). One tibial spur on L1–2 (6 specimens) or only L1 (3 specimens). The number of tarsal spurs on legs has no regularity. Two tarsal spurs on L1(2)–9 (4 specimens), L1–12(13) (3 specimens), L1–16 and L18 (1 specimen) or L1,3,5–6&8–15 (1 specimen). One tarsal spur on subsequent to penultimate legs. Ultimate legs without tarsal spur. Ultimate leg prefemur with 8–11, rarely 18 or 19 spines (2–5 ventro-lateral, 1–2 ventro-medial, 2–4 or 7 medial and 1–3 or 6 dorso-medial and 1 corner spine, rarely 2 corner spines) (Table 2).

**Table 2.** Variation in O. (O.) lewisi from Jiacha County, Xizang (0302: Ar.-MHBU-SoJC1908060302; 0303: Ar.-MHBU-SoJC1908060303; 0304 (Fig. 3): Ar.-MHBU-SoJC1908060304; DX01: Ar.-MHBU-SoJC1608DX01; 0401: Ar.-MHBU-SoJC1608120401) (L/R: Left/Right).

		Specimens				
		DX01	0302	0303	0401	0304
Length/mm		77	28	53	28	20
Number of antennal articles (L/R)		18/20	17	18/20	18	17
Number of glabrous basal antennal articles		2.25	3	2.3	3	2.2
Tooth-plate teeth		3+3	4+4*	3+3	3+3	4+4*
Paramedian sutures on tergites		4–20	3–20	3–20	6–20	4–20
Tergites marginate		7–21	4–21	8–21	3–21	5–21
Paramedian sutures on sternites		3–19	2–19	4–19 (4–9^#^)	3–19 (3–5^#^)	2–19 (2–4^#^)
Coxopleural process	Apical spines (L/R)	2	2	2	2	2
	Dorsal spines (L/R)	2	1	1/2	1	1
	Lateral spines (L/R)	2	2	2	1	1
Legs	With tibial spur	1–2	1–2	1	1	1–2
	With 1 tarsal spur	2,4,7,17,18	13–20	13–20	10–12,15–20	1,10–20
	With 2 tarsal spurs	1,3,5,6,8–15	1–12	1–12	1–9,13,14	2–9
Spines of ultimate prefemur	Ventro-lateral (L/R)	2	4/3	3	3/2	3
	Ventro-medial	1	1	1	1	1
	Medial (L/R)	2	2	3/2	2	2
	Dorso-medial (L/R)	2	3/2	2/3	2	1
	Corner spine	1	1	1	1	1
Femoral spur of leg 1		1	1	1	1	0

* Medial two teeth fused, lateral one with a small tooth on its lateral side (Fig. 3C). ^#^ Sternites with incomplete paramedian sutures.

###### Habitat.

Found under stones in arid mountain bush (Fig. 4B).

###### Distribution.

**Figure 5.**
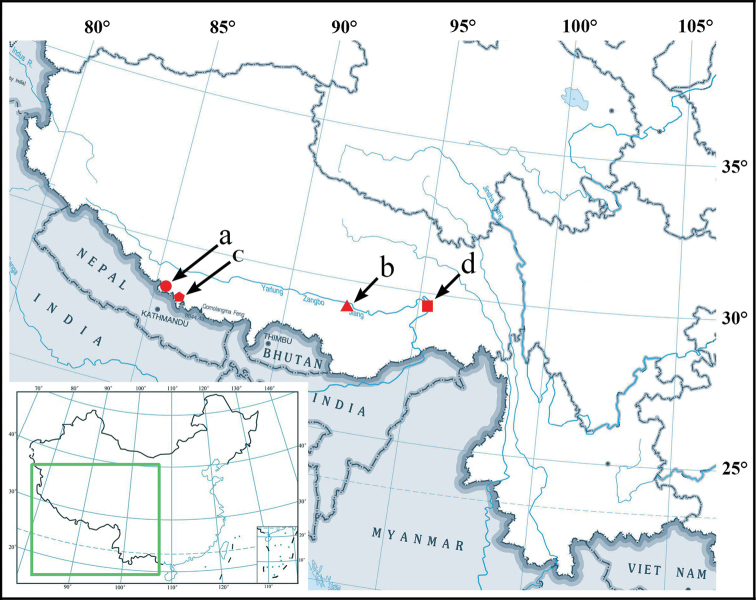
Distributions of *Otostigmus* in Xizang, China **a** circle – O. (O.) beroni, Jilong Town, Jilong (Gyirong) County **b** triangle – O. (O.) lewisi, 2005, Jiacha (Gyaca) County **c** pentagon – O. (O.) martensi, Nielamu (Nyalam) County **d** square – O. (O.) xizangensis, Bomi (Bome) County.

China (Xizang) (Fig. 5).

## ﻿Discussion

Only four species of the genus *Otostigmus*, namely, O. (O.) beroni, O. (O.) lewisi, O. (O.) martensi and O. (O.) xizangensis, were reported in Xizang, China (Fig. 5), geographically away from other species distributed throughout China. Otostigmus (O.) lewisi can be easily distinguished from O. (O.) beroni using the following characteristics: (1) larger average body length: 73 mm in O. (O.) lewisi (average of 2 adults), versus 48 mm in O. (O.) beroni (average of 5 adults); (2) body color (live): primarily brownish cephalic plate and tergites in O. (O.) lewisi, and blue-green cephalic plate and blue-brown tergites in O. (O.) beroni (Fig. 4C, D); (3) the number of teeth of tooth plates: O. (O.) lewisi 3+3 in 5 specimens or 4+4 in 4 specimens versus O. (O.) beroni 4+4 in 11 specimens or 5+5 in 6 specimens; (4) the number of tibial spurs on legs: O. (O.) lewisi with one tibial spur on legs 1–2 in 6 specimens or on legs 1 in 3 specimens. In contrast, O. (O.) beroni with one tibial spur on legs 1–4 in 8 specimens or 1–5 in 5 specimens; (5) the number of tarsal spurs on legs: O. (O.) lewisi with 2 tarsal spurs on legs 1(2)–9(13), with no regularity. In O. (O.) beroni, however, 2 tarsal spurs on legs 1–18(19); (6) the length of coxopleural process: coxopleural process of O. (O.) lewisi is shorter than that of O. (O.) beroni. The specimens described by Song et al. (2005) were immature (22–56 mm), which eventually led to a later revision by Lewis.

The most geographically close species of this genus in Xizang, China are O. (O.) beroni and O. (O.) martensi. The main difference between O. (O.) beroni and O. (O.) martensi are as follows: (1) the number of teeth of tooth plates: O. (O.) martensi 3+3, whereas, O. (O.) beroni 4+4 or 5+5; (2) the number of spines on the coxopleural process: O. (O.) martensi coxopleural process with 1 apical spine, 1 lateral spine and 1 dorsal spine. In contrast, O. (O.) beroni coxopleural process with 2–3 apical spines, 1–2 lateral spines, and 1–2 dorsal spines; (3) spurs on legs: O. (O.) martensi L1–4 or 5 with 2 tarsal spurs, whereas O. (O.) beroni L1–19 with 2 tarsal spurs, L20 with 1 tarsal spur (Lewis 2001; Song et al. 2005; Lewis 2010). Given this evidence, O. (O.) lewisi and O. (O.) martensi are obviously different species.

The distance between Jilong Town (the locality of O. (O.) beroni in China) and Zhangmu Town (Nyalam County, the locality of O. (O.) martensi in China)) is about 78 km, and the distance between Jilong Town and Jiacha County (the locality of O. (O.) lewisi in China) is about 714 km. No species of the genus *Otostigmus* was found in the area between Jilong Town (or Zhangmu Town) and Jiacha County. The habitat of O. (O.) beroni in Jilong Town is more humid than the habitat of O. (O.) lewisi in Jiacha County (Fig. 4A, B). Based on newly collected materials from Jiacha County, we reaffirm that O. (O.) lewisi is a valid species.

### ﻿Key to species of *Otostigmus* from China (followed Lewis 2001; Song et al. 2005; Niu et al. 2021)

**Table:** 

1	Ultimate sternite with sides more or less parallel	**O. (O.) astenus**
–	Ultimate sternite with sides converging posteriorly	**2**
2	Tergites typically with keel	**3**
–	Tergites typically without keel	**4**
3	Antennae with 17–19 articles	**O. (O.) xizangensis**
–	Antennae with 21 articles	**O. (O.) scaber**
4	Coxopleural process short, coxopleuron completely covered with pores	**O. (O.) politus**
–	Coxopleural process moderate or long, coxopleuron incompletely covered with pores	**5**
5	Coxopleural process with 4–7 apical spines, ultimate leg with 24–36 spines	**O. (O.) aculeatus**
–	Coxopleural process with less than 4 apical spines, ultimate leg with less than 24 spines	**6**
6	Coxopleural process with only 1 apical spine	**O. (O.) martensi**
–	Coxopleural process with 2 apical spines	**7**
7	Legs 1–4 or 5 with tibial spur, legs 1–19 with 2 tarsal spurs, leg 20 with 1 tarsal spur	**O. (O.) beroni**
–	Legs 1–2 with tibial spur, the distribution of 2 tarsal spurs in legs without regularity	**O. (O.) lewisi**

## Supplementary Material

XML Treatment for Otostigmus (O.) beroni

XML Treatment for Otostigmus (O.) lewisi
